# Psychological functioning in children suspected for mitochondrial disease: the need for care

**DOI:** 10.1186/s13023-020-1342-8

**Published:** 2020-03-24

**Authors:** Kim F. E. van de Loo, José A. E. Custers, Saskia Koene, Inge-Lot Klein, Mirian C. H. Janssen, Jan A. M. Smeitink, Christianne M. Verhaak

**Affiliations:** 1 Radboud Institute for Health Sciences, Department of Medical Psychology, Radboud Center for Mitochondrial Medicine, Amalia Children’s Hospital, Radboud University Medical Center, Geert Grooteplein Zuid 10, P.O. Box 9101, 6500HB Nijmegen, The Netherlands; 2 Radboud Institute for Health Sciences, Department of Medical Psychology, Radboud Center for Mitochondrial Medicine, Radboud University Medical Center, Nijmegen, The Netherlands; 3 Department of Pediatrics, Radboud Center for Mitochondrial Medicine, Radboud University Medical Center, Nijmegen, The Netherlands; 4 Department of Clinical Genetics, Leiden University Medical Center, Nijmegen, The Netherlands; 5 Radboud Institute for Molecular Life Sciences, Department of Internal Medicine, Radboud Center for Mitochondrial Medicine, Radboud University Medical Center, Nijmegen, The Netherlands; 6 Radboud Institute for Molecular Life Sciences, Department of Pediatrics, Radboud Center for Mitochondrial Medicine, Radboud University Medical Center, Nijmegen, The Netherlands

**Keywords:** Mitochondrial diseases, Psychology, Quality of life, Behavioral problems, Parenting stress, Diagnostic process

## Abstract

**Background:**

Mitochondrial diseases (MD) are generally serious and progressive, inherited metabolic diseases. There is a high comorbidity of anxiety and depression and limitations in daily functioning. The complexity and duration of the diagnostic process and lack of knowledge about prognosis leads to uncertainty. In this study, we investigated the psychological well-being of children who are suspected for MD and their parents.

**Methods:**

In total 122 children suspected for MD and their parents, received questionnaires as part of standard clinical investigation.

**Results:**

Parent proxy report revealed a lower quality of life (QoL) compared to norms and even more physical problems compared to chronically ill patients. They also reported more behavioral problems in general and more internalizing problems compared to the norms. Most frequent reported somatic complaints were tiredness and pain. Parents did not report enhanced levels of stress regarding parenting and experienced sufficient social support. At the end of the diagnostic process, 5.7% of the children received the genetically confirmed diagnosis of MD, 26% showed non-conclusive abnormalities in the muscle biopsy, 54% did not receive any diagnosis, and the remaining received other diagnoses. Strikingly, children without a diagnosis showed equally QoL and behavioral problems as children with a diagnosis, and even more internalizing problems.

**Conclusions:**

This study highlights the psychological concerns of children with a suspicion of MD. It is important to realize that as well as children with a confirmed diagnosis, children without a diagnosis are vulnerable since explanation for their complaints is still lacking.

## Background

Mitochondrial diseases (MD) are rare and inherited metabolic diseases, which may present with any symptom, at any age and any mode of inheritance [1]. In general, these are serious and progressive diseases, with an unpredictable disease course. The overall incidence rate is approximately 1:5000 live births [2, 3]. Mitochondria play an important role in the energy production of the cell. Organs with the highest energy requirement, like the brain and the muscles, are most likely to be affected [4]. There is a wide variety in genetic and biochemical involvement as well as in phenotypic expression [5]. MD could be caused by a mutation in the mitochondrial DNA (mtDNA) or nuclear DNA (nDNA). The diagnosis of MD is acquired through multiple steps; the clinical presentation (complaints, signs and symptoms, inheritance), clinical chemistry, metabolic studies, pathological evaluation, biochemical and genetic testing [6]. To date, no cure is available.

The impact of having MD is major for the child and his/her parents. The most burdensome complaints in children are fatigue, behavior and speech disturbances, epilepsy and muscle weakness, and a high degree of limitations in daily activities [1]. Furthermore, anxiety and depression are common in children with MD [7, 8], with an even higher comorbidity compared to children with other types of inborn errors of metabolism and compared to patients with Sotos syndrome [8]. The severe limitations children experience and the large variability in clinical manifestations also result in a high impact on the well-being of the caregiver [9]. Parents often experience stress and worries [10, 11]. Parenting stress is higher when there are more hospitalizations and increased use of special services, and when there is more organ involvement [11].

It seems clear that the psychological impact of having MD is high. Less is known about the psychological functioning of children and their parents who are in the diagnostic process. The diagnostic process is complex and may take years between onset of first symptoms and confirmed diagnosis [12]. This lengthy time as well as a lack of knowledge about the diagnosis leads to uncertainty and stress [12]. In children and their parents, to our knowledge, no research exists addressing the psychological well-being during the diagnostic process. The primary objective of the current study is to investigate the psychological well-being of children who are suspected for MD and their parents.

## Methods

### Participants

All parents of children (< 18 years) suspected for MD, who received a muscle biopsy at the Radboud Center for Mitochondrial Medicine (RCMM), Radboudumc Amalia Children’s Hospital in Nijmegen (the Netherlands) between January 2010 and April 2019 were included. Indication for a muscle biopsy was determined based on international guidelines at time of assessment.

### Procedure

Parents received questionnaires as part of a standard healthcare program before muscle biopsy. This program was started in January 2010 and inclusion ended in April 2019. Parents had to be fluent in Dutch in order to understand the questionnaires. After completion of the questionnaires, the results of the final diagnosis (based on muscle biopsy studies, mtDNA and/ or whole exome sequencing), were also investigated.

### Materials

#### Demographic factors and disease characteristics

Demographic and disease characteristics, like duration of health complaints and involved specialists, were assessed by a 20-item questionnaire filled in by one parent of every child.

#### Child outcome measures

Quality of life (QoL) was assessed with the Pediatric Quality of Life Inventory (PedsQL) [13]. Parent proxy report (children aged 5 to 18 years) was used. A higher score indicates a better health-related QoL [13].

Behavioral problems of the child were assessed with the Child Behavior Checklist (CBCL) (parent-reported questionnaire) [14, 15]. The CBCL provides scores on global, internalizing and externalizing behavioral problems. The CBCL is divided into two age categories: 1,5 to 5 years and 6 to 18 years. Available norms provide age and gender-standardized T-scores (*M* = 50; *SD* = 10). Total, internalizing and externalizing T-scores ≥60 and syndrome scale T-scores ≥65 represent the borderline, whereas scale T-scores ≥64 and syndrome scale T-scores ≥70 represent the clinical range in the general population [14, 15].

#### Parental outcome measures

Parenting stress was assessed with the Parenting Stress index (PSI), short version (Nijmeegse Ouderlijke Stress Index NOSIK) [16]. This questionnaire evaluates the stress parents experience in raising their child in the age of 1 to 13 years. Scores were rated into normal (score mothers < 74; score fathers < 64), subclinical (score mothers 74–89; score fathers 64–78) and clinical levels of parenting stress (score mothers > 89; score fathers > 78).

Parental perceived social support was assessed with the Inventory for Social Reliance (ISR) [17]. The ISR evaluates the social support network of parents of children in the age from 0 to 18 years. The total score was used as a measure of experienced social support. Scores above 10 were rated as normal/ sufficient amount of social support, 7–9 indicated a low amount, and 6 or below were seen as insufficient amount of social support [18].

### Statistical analysis

All statistical analyses were conducted by using the Statistical Package for Social Sciences (SPSS) for Windows version 25.0 (IBM Corp., Armonk, NY). Normal distribution of continuous data was assessed using Shapiro-Wilk test of normality.

Parent report scores of the PedsQL were compared, using one sample t-tests, to the norm scores of a healthy population sample and a general chronic health condition sample, defined in Varni et al. (2006) as “*a physical or mental health condition that has lasted or is expected to last at least 6 months and interferes with the child’s activities”* [19].

The parent proxy report of behavioral problems (CBCL) was assessed with different age versions. Since subscales of both versions using similar concepts, we used the sum score of each concept for calculations when possible. The percentage of behavioral problems above the clinical cutoff were calculated. The total, internalizing and externalizing problems were compared to the norms [14, 15]. Assessment of depression in a somatic population is difficult as vital aspects of depression are also apparent as symptoms of somatic conditions (e.g. less active). Following assessment of depression in adult populations [20], we deleted items relating to vital functioning in the assessment of depression. We used both the original scale (CBCL withdrawn/depressed), as well as an adjusted subscale (CBCL withdrawn/depressed_adj_), in which the somatic items are replaced by the mean score of the remaining subscale items. Specific items of the somatic complaints which could also be core symptoms of MD were investigated by using percentages of item scores > 2. Chi-square tests, linear-by-linear association, were used to investigate differences between children with or without a diagnosis in rated items (0,1,2) of somatic complaints. As an indication of possible not met needs we investigated how many children with behavioral problems (clinical score on the total, internalizing or externalizing scale), had support from a dedicated specialist, psychologist or remedial educationalist.

Differences between children with (MD, uncertain abnormalities or other diagnoses) and without a diagnosis were investigated by using independent samples t-test or in case of non-normal distribution, a Mann-Whitney U test. Variables of interest (mothers report of the PedsQL Total scale, CBCL Total, Internalizing, Externalizing, Anxious/Depressed, Withdrawn/Depressed_(adj)_, Somatic complaints and both PSI and ISR of fathers and mothers) were used for analyses.

## Results

### Demographics and disease characteristics

In total 122 children and their parents participated (Table 1). The majority of children perceived health complaints for more than 3 years. In almost 75% of the children, one or more specialist(s) were involved. Most frequently involved were physiotherapy (55%), followed by speech therapy and a dietician (both 28%). A psychologist or remedial educationalist were involved in respectively 17 and 11% of all children.

**Table 1:** Demographic variables and disease/ health related characteristics

Demographics	Children N (%, Total)	Family/ general information	Mothers	Fathers
Mean age	8.4 years (0–17)		38.7 (25–51)	41.0 (24–54)
Boys	67 (54.9%, 122)			
Child living at home	115 (100%)			
School/childcare				
• *Non*	9 (7.8%, 115)			
• *Regular*	85 (73.9%)			
• *Special*	21 (18.3%)			
Nationality *Dutch*			107 (97.3%, 110)	94 (92.2%, 102)
Level of education:				
• *Elementary school*			0 (0%, 108)	3 (2.9%, 105)
• *Secondary education*			13 (12.0%)	8 (7.6%)
• *Lower vocational education*			7 (6.5%)	9 (8.6%)
• *Intermediate vocational education*			56 (51.9%)	52 (49.5%)
• *Higher vocational education*			26 (24.1%)	19 (18.1%)
• *University*			6 (5.6%)	14 (13.3%)
Having a job			72 (65.5%, 110)	95 (91.3%, 104)
Marital status:				
• *Married*		87 (76.3%, 114)		
• *Living together*		18 (15.8%)		
• *Single parent*		6 (5.3%)		
• *Divorced*		3 (2.6%)		
**Disease/ health related characteristics**				
Duration health complaints				
• *< 1 year*	11 (9.8%, 112)			
• *1–2 years*	18 (16.1%)			
• *> 3 years*	83 (74.1%)			
Treatment in other care instances	87 (75.0%, 116)			
Involvement of other specialists in total	85 (74.6%, 114)			
• *Speech therapy*	32 (28.1%, 114)			
• *Physiotherapy*	63 (55.3%, 114)			
• *Psychologist*	19 (16.7%, 114)			
• *Remedial educationalist*	13 (11.4%, 114)			
• *Social worker*	12 (10.5%, 114)			
• *Dietician*	32 (28.1%, 114)			
• *Occupational therapist*	16 (14.0%, 114)			
Care leave			59 (56.7%, 104)	65 (67.7%, 96)
Health problems: *In family*		73 (63.5%, 115)		
• *Mother/Father*			46 (43.8%, 105)	20 (19.8%, 101)
• *Brothers/sisters*		43 (41.3%, 104)		
Other concerns in general			22 (20.8%, 106)	11 (12.9%, 85)
Diagnoses				
• *No diagnosis*	67 (54.9%)			
• *Mitochondrial disease*	7 (5.7%)			
• *Non-conclusive abnormalities*	32 (26.2%)			
• *Confirmed other diagnosis*	15 (12.3%)			
• *No diagnosis up to date*	1 (0.8%)			

Results from the muscle biopsy and further diagnostics (genetics) show that 54.9% of all children did not receive any diagnosis, and only 5.7% received the diagnosis of having MD. 26.2% of the children showed at the time non-conclusive abnormalities in the muscle biopsy and genetic analysis. The remaining 12.3% received other genetic diagnoses, and in of one patient there is no final diagnosis up to date.

### Child outcomes

#### Quality of life

Parent proxy-report revealed a lower QoL in total and on all subscales compared to the general population (Table 2). Compared to other patients with a chronic health condition, fathers and mothers also reported more problems with physical functioning in their child and mothers also a lower QoL in general. In contrast, fathers reported a better social functioning for their children compared to other patients with a chronic health condition.

**Table 2:** Child’s quality of life as reported by their parents (PedsQL)

Quality of Life (PedsQL)	N*	Mean (SD)	Norms healthy population^**a**^	Student’s t	Norms chronic ill population^**a**^	Student’s t
***Parent proxy report- Mother***						
Total	84	59.57 (16.23)	77.61	T(83) = −10.19, *p* = .000	64.05	T(83) = −2.53, *p* = .013
Physical	84	47.89 (24.60)	79.20	T(83) = −11.66, *p* = .000	66.38	T(83) = −6.89, *p* = .000
Emotional	84	68.87 (20.01)	77.65	T(83) = −4.02, *p* = .000	64.85	T(83) = 1.84, *p* = .069
Social	84	67.26 (18.41)	79.51	T(83) = − 6.10, *p* = .000	63.45	T(83) = 1.90, *p* = .061
School	83	61.34 (16.40)	73.12	T(82) = −6.54, *p* = .000	60.36	T(82) = .55, *p* = .588
Psychosocial	84	65.83 (14.47)	76.76	T(83) = −6.92, *p* = .000	62.87	T(83) = 1.88, *p* = .064
***Parent proxy report- Father***						
Total	62	60.12 (16.87)	77.61	T(61) = −8.16, *p* = .000	64.05	T(61) = −1.83, *p* = .071
Physical	62	48.27 (24.98)	79.20	T(61) = −9.75, *p* = .000	66.38	T(61) = −5.71, *p* = .000
Emotional	63	69.52 (19.81)	77.65	T(62) = −3.26, *p* = .002	64.85	T(62) = 1.87, *p* = .066
Social	61	68.87 (17.28)	79.51	T(61) = −4.85, *p* = .000	63.45	T(61) = 2.47, *p* = .016
School	61	61.13 (16.43)	73.12	T(60) = −5.70, *p* = .000	60.36	T(60) = .37, *p* = .717
Psychosocial	63	66.53 (14.73)	76.76	T(62) = −5.51, *p* = .000	62.87	T(62) = 1.97, *p* = .053

* Of the 89 children who were 5 years or older, 84 parents filled in the PedsQL. Mother report only: *N* = 22, father report only: *N* = 0, mother and father report: *N* = 62

^a^Norms Varni et al., 2006 (the pedsql as a population health measure)

#### Child behavior

Mean scores, standard deviations, as well as percentage scoring in the clinical range of the CBCL are described in Table 3. Mothers and fathers both reported higher scores on the total scale (Mothers X^2^ [1]=12.67, *p* = .000, fathers X^2^ [1]=8.65, *p* = .003), and more internalizing problems (Mothers X^2^ [1]=138.72, *p* = .000, fathers X^2^ [1]=57.32, *p* = .000) in the clinical range compared to the norms.

**Table 3:** Child’s behavioral problems as reported by their parents (CBCL)

Scale/subscale	Mothers report		Father report		Diagnosis vs no diagnosis, mothers report
	*Mean T-score (SD) N = 113–115*	*% in clinical range*	*Mean T-score (SD) N = 82–84*	*% in clinical range*	
Total score	55.91 (10.04)	18.6**	54.15 (10.64)	18.3*	T(110) = 1.64, *p* = .104
Internalizing	59.35 (11.27)	40.7**	57.71 (11.58)	32.9**	T(110) = 2.36, *p* = .020^d^
Externalizing	48.36 (10.08)	7.9	47.84 (9.88)	7.2	T(111) = −.24, *p* = .811
Anxious/depressed	55.66 (8.08)	5.2	55.00 (7.52)	3.6	U = 1276, *p* = .069
Somatic complaints	65.44 (10.37)	36.8	63.40 (11.22)	25.3	T(111) = 1.49, *p* = .140
*Pain*^*c*^		*34.2*		*27.7*	χ^2^(1) = 1.21, *p* = .272
*Tiredness*^*cb*^		*51.9*		*40.7*	χ^2^(1) = 492, *p* = .483
*Headache*^*c*^		*16.1*		*14.5*	χ^2^(1) = 4.12, *p* = .042^d^
*Stomach pain/cramps*^*c*^		*14.2*		*11*	χ^2^(1) = 1.19, *p* = .276
*Obstipation*^*c*^		*9.6*		*11.9*	χ^2^(1) = .45, *p* = .503
*Doesn’t eat well*^*ca*^		*25.0*		*26.7*	χ^2^(1) = .40, *p* = .530
Withdrawn/depressed	59.01 (8.47)	12.3	57.77 (7.74)	13.3	T(111) = 1.71, *p* = .090
Withdrawn/depressed_adj_	56.34 (7.54)	7.0	55.65 (6.91)	4.8	T(111) = .62, *p* = .535
Attention problems	58.26 (8.26)	8.8	57.67 (8.80)	8.4	
Aggressive behavior	53.69 (6.69)	3.5	53.29 (5.97)	2.4	
Emotional reactive (1.5–5)^a^	56.86 (9.96)	8.6	56.83 (6.27)	0	
Sleep problems (1.5–5)^a^	55.23 (6.85)	2.9	52.86 (4.76)	0	
Social problems (6–18)^b^	57.80 (7.68)	5.1	57.17 (8.15)	9.3	
Thought problems (6–18)^b^	58.13 (9.21)	12.7	58.56 (9.46)	16.7	
Rule breaking behavior (6–18) ^b^	52.33 (4.12)	1.3	52.54 (4.30)	1.9	

*P < .05

**P < .001

^a^Version 1.5–5 years, *N* = 35 mothers, 29 fathers

^b^Version 6–18 years, *N* = 79 mothers, 54 fathers

^c^Item of the subscale somatic complaints. Rated as percentage of scores > 2 (‘very often/ true’ as response)

^d^patients without a diagnosis have a higher mean score compared to patients with a diagnosis

A total of 12.3% of the children show withdrawn/depressed behavior according to their mothers and 13.3% as reported by their fathers. When adjusting for ambiguous items which both load on withdrawn/depressed as well as disease symptoms, child’s behavior was rated as withdrawn/depressed in 7.0% (mothers report) and 4.8% (fathers report) of the children. Scores of the withdrawn/depressed adjusted scale were significantly lower, indicating less concerns, after correcting for the somatic items in both mothers (t(113) = − 9.57, *p* = .000) and fathers report (t(82) = − 7.11, *p* = .000).

Most frequent reported somatic complaints were tiredness (mothers report: 51.9%, fathers report: 40.7%) and pain (mothers report: 34.2%, fathers report: 27.7%).

In total, 52 of the 113 children showed behavioral problems on one of the scales (total, internalizing or externalizing), of which 9 (17%) received help from a psychologist or remedial educationalist.

### Parental outcome measures

#### Parenting stress

Compared to a clinical population, both fathers and mothers reported less parenting stress. Mothers also reported less parenting stress compared to the non-clinical population. There was no significant difference between fathers and mothers in parenting stress (t(67) = − 0.46, *p* = .649). In total 5.9% of the mothers experienced parenting stress in the clinical range and 11.1% of the fathers (Table 4).

**Table 4:** Parental outcome measures: parenting stress (PSI) and social support (ISR)

	N	Mean (SD)	% in clinical range	Non-clinical population	Student’s t	Clinical population	Student’s t
**PSI**							
Mothers	85	47.80 (20.23)	5.9%	54.4^a^	T(84) = −3.009*	85.9^a^	T(84) = −17.368**
Fathers	72	49.10 (20.89)	11.1%	48.5^a^	T(71) = .243	70.4^a^	T(71) = −8.653**
**ISR**							
Mothers	111	17.01 (3.79)	1.8%	15.1^b^	T(110) = 5.302**	14.5^c^	Wilcoxon, *p* = .000
Fathers	89	16.82 (3.76)	2.2%	15.1^b^	T(88) = 4.318**	14.5^c^	Wilcoxon, *p* = .000

*P < .05

**P < .001

^a^Norms De Brock 1992

^b^Norms van Dam-Baggen and Kraaijmaat (1992)

^c^Huiskes Kraaijmaat Bijlsma 2004

#### Social support

Both mothers and fathers experienced more social support compared to the norms [17] and more social support compared to patients with rheumatoid arthritis [18] (Table 4). In total, 98.2% of the mothers and 97.8% of the fathers experienced sufficient social support.

### Comparison children with and without a diagnosis in psychological functioning

There was no difference between children with any diagnosis or without a diagnosis in QoL (mothers report: t(81) = −.806, *p* = .422) or in total or external behavioral problems (see Table 3). Regarding internal behavioral problems, mothers reported more problems of their children without a diagnosis (mean T-score = 61.35) compared to children with a diagnosis (mean T-score = 56.36). There were no differences between children with or without a diagnosis in the amount of reported somatic complaints, except for headache. Mothers of children without a diagnosis report more complaints of headache in their child compared to children with a diagnosis.

There were no significant differences between the groups with or without a diagnosis in parenting stress (mothers t(82) = −.626, *p* = .533, fathers t(69) = .246, *p* = .806), and social support (mothers t(108) = −.447, *p* = .665, fathers t(86) = 1.134, *p* = .260).

## Discussion

To the best of our knowledge, this is the first study investigating the psychological well-being of children who are in the diagnostic process of a suspected MD, and their parents. Overall, results showed substantial problems in child psychological functioning, while parents do not report enhanced levels of parenting stress or a lack of social support.

Parent proxy-report revealed a lower QoL on all areas compared to the norms, which is in line with adult studies of patients with proven MD [21, 22]. Children also showed more problems with physical functioning compared to children with other general chronic illnesses, indicating serious impairments.

Regarding behavioral problems, compared to norms, parents reported more problems in general and more internalizing problems specifically in their child, which is in line with other literature in children with chronic diseases [23]. Although both the CBCL total and internalizing scale are including somatic items, a meta-analysis [23] showed that elevated levels of both scales remained after controlling for confounding aspects of items related to somatic condition. Surprisingly, children without a diagnosis showed more internalizing problems compared to children with a diagnosis, indicating a high need for help in these children. Based on results of this study, we cannot explain these findings. The underlying mechanisms for behavioral problems may be different in these groups and should be investigated in future research.

As mentioned before, anxiety and depression are common in children with MD [7, 8] suggesting a possible link with abnormal central nervous system energy metabolism [7]. This study also showed a high percentage of anxious/depressed behavior and withdrawn/depressed behavior. After correcting for possible somatic symptoms, the percentage of experienced withdrawn/depressed complaints dropped significantly. Furthermore, we did not find any differences between children with or without a diagnosis. An explanation could be that the withdrawn/ depressed complaints may not solely be inherent to the disease, but could also be correlated to other factors, for instance inherent to a long history of experiencing specific somatic complaints.

It is known that the somatic complaints subscale of the CBCL is difficult to interpret in children with chronic disease, since items could overlap with illness specific symptoms [23, 24]. On item-level, results revealed a remarkable high percentage of tiredness and pain in children suspected for MD, which are also core symptoms of the disease. There was no difference in these items between children with a diagnosis compared to children without a diagnosis. Surprisingly, children without a diagnosis more frequently reported complaints of headache, indicating debilitating somatic problems in this group.

Contradictory to what was expected [10, 11], the majority of the parents did not report parenting stress and mothers reported even less parenting stress compared to the general population. Although there was no difference found between parents of children with or without a diagnosis, this study examined parenting stress before diagnosis, instead of afterwards. A diagnosis of MD brings along, e.g. more hospitalizations, and an increased use of special healthcare services, which is correlated with higher parenting stress [12]. It is important to note that the measurements and thereby the concepts of parenting stress were different between studies. Another explanation could be that parents in our study experienced sufficient social support from their environment, which is seen as a healthy coping behavior, related to less parenting stress [11]. Finally, being in the diagnostic phase could also influence the amount of reported concerns. Possibly the child’s well-being is the pith of the matter at time of assessment and parents interpret their child’s behavior in light of the possible disease and therefore not so much as ‘stressful’ but rather in light of compassion and sympathy. It would be interesting to monitor parenting stress after the diagnostic process, especially when there is no diagnosis, to investigate the impact of the possible disease on the perception of parenting stress.

In this cohort of patients suspected for MD, 7 out of the 122 children received a genetically confirmed diagnosis while 26% of the children showed non-conclusive abnormalities in the muscle biopsy without a yet confirmed genetic mutation. When interpreting these results, it is important to keep in mind that this study started in 2011, and since then the available genetic knowledge and diagnostic tools for mitochondrial disease have markedly improved. Despite this, there are still several limitations in the diagnostic steps required for a diagnosis, amongst which is a lack of understanding of the role of the entire genome in mitochondrial function [25]. We expect that in some of the children with observed non-conclusive abnormalities in the muscle biopsy, future studies will reveal pathogenic mutations. However, for others these might not be discovered due to e.g. non-genetic external factors hampering proper mitochondrial functioning like muscle disuse.

At the end of the diagnostic process, not limited to mitochondrial disease only, more than half of the patients did not receive any diagnosis explaining their debilitating complaints. Given the fact that we found that these children experience the same amount of psychological problems and physical complaints as children with a diagnosis, and even more concerns regarding internalizing problems and headache, these children are especially vulnerable. In the Netherlands these patients do not have automatically the same access to care as patients with a confirmed diagnosis, since they do not fit in a standard care program. For these children, we strongly recommend screening for psychological problems and provide access to care they need.

Having a child with serious complaints that are unexplained confronts parents of children with a rare disease with serious uncertainty. Uncertainty regarding medical conditions especially exists in situations that are ambiguous, complex and unpredictable [26], all applicable to the complaints of children in this study. A lack of diagnosis leaves patients and parents with even more uncertainty. Studies on the impact of rare diseases on children and their parents stress the impact of the long diagnostic process and the diagnostic delays [27, 28]. This long period could seriously impact on health of children and their families. Not only because of possible medical health care needs stay unmet, also the impact of uncertainty regarding the child’s health and the look for diagnostics and adequate care means a serious burden. Health care providers could guide parents through possible additional diagnostic trajectories, and by supporting them in dealing with uncertainty, and focus on aspects of their life they can control. This paper stresses the importance of keeping these children and their parents in our focus.

When investigating the met needs of all included children, with or without a diagnosis, only 17% of the children with behavioral problems received help from a specialist on child behavior, leaving out 73% with a need for help. By including assessment on psychological health, preferably by standardized questionnaires and a psychologist in diagnostic procedures, adequate help can be provided in an early phase.

When interpreting the above-mentioned results, it is important to take note of the strengths and limitations. A strength is the relatively large sample size. Secondly, by screening in the beginning of the diagnostic process, before diagnosis, perceived complaints were not influenced by knowing if they did or did not have the disease. A limitation is the large time span of this study, since diagnostic criteria and possibilities have been improved. Furthermore, due to age limitations of various questionnaires, not all patients and parents filled in the same questionnaires. Finally, since all patients filled in questionnaires as part of standard care, no information regarding e.g. fatigue in children aged < 6 years, was available, which is the core complaint of MD in children [1].

## Conclusions

This study highlights the psychological concerns of children with a suspicion of MD. It is important to be aware of these problems in an early stage and to provide adequate help, independent of the final diagnosis. Especially the children without any diagnosis confirmed are vulnerable since they remain in uncertainty and do not fit in a standard special care program.

## Data Availability

The dataset used during the current study is available from the corresponding author on reasonable request.

## Abbreviations

CBCL: Child Behavior Checklist
ISR: Inventory for Social Reliance
MD: Mitochondrial diseases
mtDNA: Mitochondrial DNA
nDNA: Nuclear DNA
NOSIK: Nijmeegse Ouderlijke Stress Index
PedsQL: Pediatric Quality of Life Inventory
QoL: Quality of life
RCMM: Radboud Center for Mitochondrial Medicine
SPSS: Statistical Package for Social Sciences
